# Changes in the sensitivity of GABA_A_ current rundown to drug treatments in a model of temporal lobe epilepsy

**DOI:** 10.3389/fncel.2013.00108

**Published:** 2013-07-11

**Authors:** Pierangelo Cifelli, Eleonora Palma, Cristina Roseti, Gianluca Verlengia, Michele Simonato

**Affiliations:** ^1^Section of Pharmacology, Department of Medical Sciences, University of FerraraFerrara, Italy; ^2^National Institute of NeuroscienceFerrara, Italy; ^3^Ri.MED FoundationPalermo, Italy; ^4^Istituto di Ricovero e Cura a Carattere Scientifico “San Raffaele Pisana”Rome, Italy; ^5^Dipartimento di Fisiologia e Farmacologia and Istituto Pasteur-Fondazione Cenci BolognettiRome, Italy

**Keywords:** pilocarpine, GABA, hippocampus, neocortex, BDNF, levetiracetam

## Abstract

The pharmacological treatment of mesial temporal lobe epilepsy (mTLE), the most common epileptic syndrome in adults, is still unsatisfactory, as one-third of the patients are or become refractory to antiepileptic agents. Refractoriness may depend upon drug-induced alterations, but the disease *per se* may also undergo a progressive evolution that affects the sensitivity to drugs. mTLE has been shown to be associated with a dysfunction of the inhibitory signaling mediated by GABA_A_ receptors. In particular, the repetitive activation of GABA_A_ receptors produces a use-dependent decrease (rundown) of the evoked currents (*I*_GABA_), which is markedly enhanced in the hippocampus and cortex of drug-resistant mTLE patients. This phenomenon has been also observed in the pilocarpine model, where the increased *I*_GABA_ rundown is observed in the hippocampus at the time of the first spontaneous seizure, then extends to the cortex and remains constant in the chronic phase of the disease. Here, we examined the sensitivity of *I*_GABA_ to pharmacological modulation. We focused on the antiepileptic agent levetiracetam (LEV) and on the neurotrophin brain-derived neurotrophic factor (BDNF), which were previously reported to attenuate mTLE-induced increased rundown in the chronic human tissue. In the pilocarpine model, BDNF displayed a paramount effect, decreasing rundown in the hippocampus at the time of the first seizure, as well as in the hippocampus and cortex in the chronic period. In contrast, LEV did not affect rundown in the hippocampus, but attenuated it in the cortex. Interestingly, this effect of LEV was also observed on the still unaltered rundown observed in the cortex at the time of the first spontaneous seizure. These data suggest that the sensitivity of GABA_A_ receptors to pharmacological interventions undergoes changes during the natural history of mTLE, implicating that the site of seizure initiation and the timing of treatment may highly affect the therapeutic outcome.

## INTRODUCTION

Mesial temporal lobe epilepsy (mTLE) is the most common form of epilepsy of adulthood. In mTLE an initial “epileptogenic event” (head trauma, stroke, brain infection or tumor) is often identifiable which is followed, after a latent period of weeks to years, by the spontaneous occurrence of seizures. Multiple pathological and physio-pathological alterations have been identified that may be responsible for the transformation of a normal brain in an epileptic one (Pitkänen and Lukasiuk, 2011). In particular, we have focused on alterations in the GABA system, and found that GABA_A_ receptors from epileptic tissue (hippocampus and neocortex) become less responsive to repeated activation (as detected by current rundown) than those from healthy tissue (Palma et al., 2004, 2007a,b; Ragozzino et al., 2005). This use-dependent GABA_A_ receptor desensitization may imply hyper-excitability and favor the occurrence of spontaneous seizures. This phenomenon occurs both in human tissue and in animal models (pilocarpine), becomes detectable in the hippocampus at the time of the first spontaneous seizure and may depend upon alteration in the molecular composition of the GABA_A_ receptor (Mazzuferi et al., 2010).

Once spontaneous seizures begin to occur and the diagnosis of epilepsy is made, the disease often continues to progress, with increasing severity of seizures; neurological decline and appearance of co-morbidities; development of resistance to pharmacological treatments (Pitkänen and Sutula, 2002). Many studies have been performed that provided mechanistic interpretations for the development of pharmaco-resistance. The best-described mechanisms are drug-related, i.e., drug-induced alterations in transport to the CNS (blood–brain barrier crossing) or in pharmacodynamics, which lead to attenuation or loss of therapeutic effects (Schmidt and Löscher, 2005). However, the progression of the disease *per se* may also implicate alterations in the responsiveness to pharmacological agents. Identifying these disease-induced alterations in the response to drugs may provide the basis for more effective treatment strategies in the different phases of mTLE.

To challenge the hypothesis that the disease progression affects drug responsiveness, we explored the sensitivity to pharmacological treatments of the increased rundown of the GABA current (*I*_GABA_) observed in epileptic tissue at different stages of experimental mTLE, namely at the time of the first spontaneous seizure and in the chronic period. We employed two structurally unrelated agents, levetiracetam (LEV) and brain-derived neurotrophic factor (BDNF), because both have been previously demonstrated to be capable of reducing the increased *I*_GABA_ rundown in the human and also in the rat epileptic brain (Palma et al., 2005b, 2007a,b). Whereas LEV is a clinically employed anti-epileptic drug, BDNF has been reported to provide anti-epileptic effects under some (Paradiso et al., 2009) but not all (He et al., 2004) experimental conditions and is not in clinical use.

## MATERIALS AND METHODS

### ANIMALS

Male Sprague-Dawley rats (240–260 g; Harlan, Italy) were used for all experiments. Animals were housed under standard conditions: constant temperature (22–24°C) and humidity (55–65%), 12-h dark–light cycle, and free access to food and water. All efforts were made to minimize animal suffering. Procedures involving animals and their care were carried out in accordance with European Community and national laws and policies (authorization number: D.M. 83/2009-B; 246/2012-13).

### PILOCARPINE

Pilocarpine was administered i.p. (300 mg/kg), and behavior was observed for several hours thereafter. Within the first hour after injection, all animals developed seizures evolving into recurrent generalized convulsions [status epilepticus (SE); average time between pilocarpine administration and onset of convulsive SE: 19 ± 2]. SE was interrupted 3 h after onset by administration of diazepam (10 mg/kg i.p). The animals were then assigned to two experimental groups representing different phases of the natural history of the disease: a subgroup was sacrificed 6 h after the first spontaneous seizure; the other subgroup was sacrificed 1 month after SE, i.e., in the chronic period when animals were experiencing an average of 5.3 ± 1.2 spontaneous seizures per day.

Seizures were assessed by 24/24-h, 7/7-day video monitoring, performed using a digital video surveillance system DSS1000 (AverMedia Technologies, USA). Recording electrodes were implanted in the hippocampus and cortex for identification of the first spontaneous seizure [continuous video-EEG (electroencephalogram) monitoring from day 4 after SE until the day of the first spontaneous seizure]. EEG seizure were categorized as paroxysmal activity of high frequency (>5 Hz) characterized by a >3-fold amplitude increment over baseline (Williams et al., 2009; Paradiso et al., 2011). Seizure severity was scored using the scale of Racine (1972): (1) chewing or mouth and facial movements; (2) head nodding; (3) forelimb clonus; (4) generalized seizure with rearing; (5) generalized seizure with rearing and falling. Analysis was performed by two independent investigators that were blind for the group to which the rats belonged. In case of differential evaluation, data were reviewed together to reach a consensus (Paradiso et al., 2011). In the chronic period, animals were continuously video recorded for a week before being killed (i.e., 23–30 days after SE), to identify frequency and duration of generalized seizures.

### OOCYTES

Membranes were prepared from the hippocampus and the fronto-temporal cortex. Preparation of *Xenopus laevis* oocytes and injection procedures were as previously described in detail (Miledi et al., 2006). Briefly, tissues were homogenized using a Teflon glass homogenizer with 2 ml of assay buffer of the following composition (in mM): 200 glycine, 150 NaCl, 50 ethylene glycol tetraacetic acid (EGTA), 50 ethylenediaminetetraacetic acid (EDTA), 300 sucrose; 20 μl protease inhibitors (Sigma Aldrich Inc., USA); pH 9 (adjusted using NaOH). The homogenate was centrifuged for 15 min at 9,500 *g*. The supernatant was collected and centrifuged for 2 h at 105 *g* at 4°C. The pellet was washed, re-suspended in 5 mM glycine and used directly, or aliquoted and stored at -80°C for later use. From 12 to 48 h after injection, membrane currents were recorded from voltage-clamped oocytes using two microelectrodes filled with 3 M KCl. The oocytes were placed in a recording chamber (0.1 ml) perfused continuously (9–10 ml/min) with oocyte’s Ringer solution (OR) at room temperature (20–22°C). OR had the following composition (in mM): NaCl 82.5, KCl 2.5, CaCl_2_ 2.5, MgCl_2_ 1, 4-(2-hydroxyethyl)-1-piperazineethanesulfonic acid (HEPES) 5, pH 7.4 (adjusted using NaOH). GABA current rundown was defined as the decrease (in percentage) of the current peak amplitude after six 10-s applications of 1 mM GABA at 40-s intervals (Palma et al., 2007a). The fast *I*_GABA_ desensitization was defined as the time taken for the current to decay from its peak to half-peak value (*T*_0.5_).

Levetiracetam was dissolved in H_2_O and stored as frozen stock solutions (100 mM). BDNF (Sigma) was dissolved in H_2_O, stored as frozen stock solutions (50 μg/ml). Both LEV and BDNF were diluted to working concentrations shortly before the experiments and applied to oocytes for 2 h. In all experiments the holding potential was -60 mV. In some experiments, 3 h washout with OR was performed before initiation of a new rundown protocol.

**FIGURE 1:**
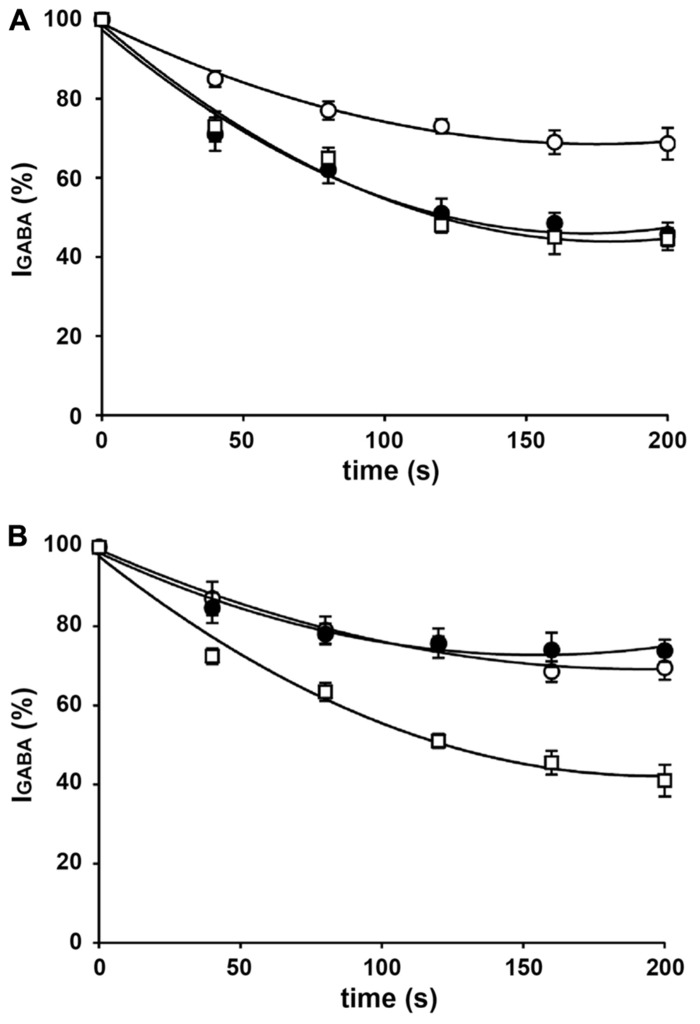
***I*_GABA_ rundown in oocytes injected with rat brain membranes.** **(A)** Time course of current rundown in oocytes injected with hippocampal membranes form control rats (◦), rats killed after the first spontaneous seizure (•), and rats killed in the chronic period, 30 days after SE (□). Peak amplitudes of *I*_GABA_ were normalized to those elicited by the first GABA application (95 ± 20 nA in the control group; 115 ± 21 nA in the first seizure group; 98 ± 18 nA in the chronic group). **(B)** Time course of current rundown in oocytes injected with cortical membranes form control rats (◦), rats killed after the first spontaneous seizure (•), and rats killed in the chronic period, 30 days after SE (□). Peak amplitudes of *I*_GABA_ were normalized to those elicited by the first application (190 ± 25 nA in control; 230 ± 25 nA in first seizure; 201 ± 30 nA in chronic). GABA pulses were 1 mM, 10-s duration every 40 s. Data are means ± SEM (*n* = 12). All data sets fitted to an exponential curve.

**FIGURE 2:**
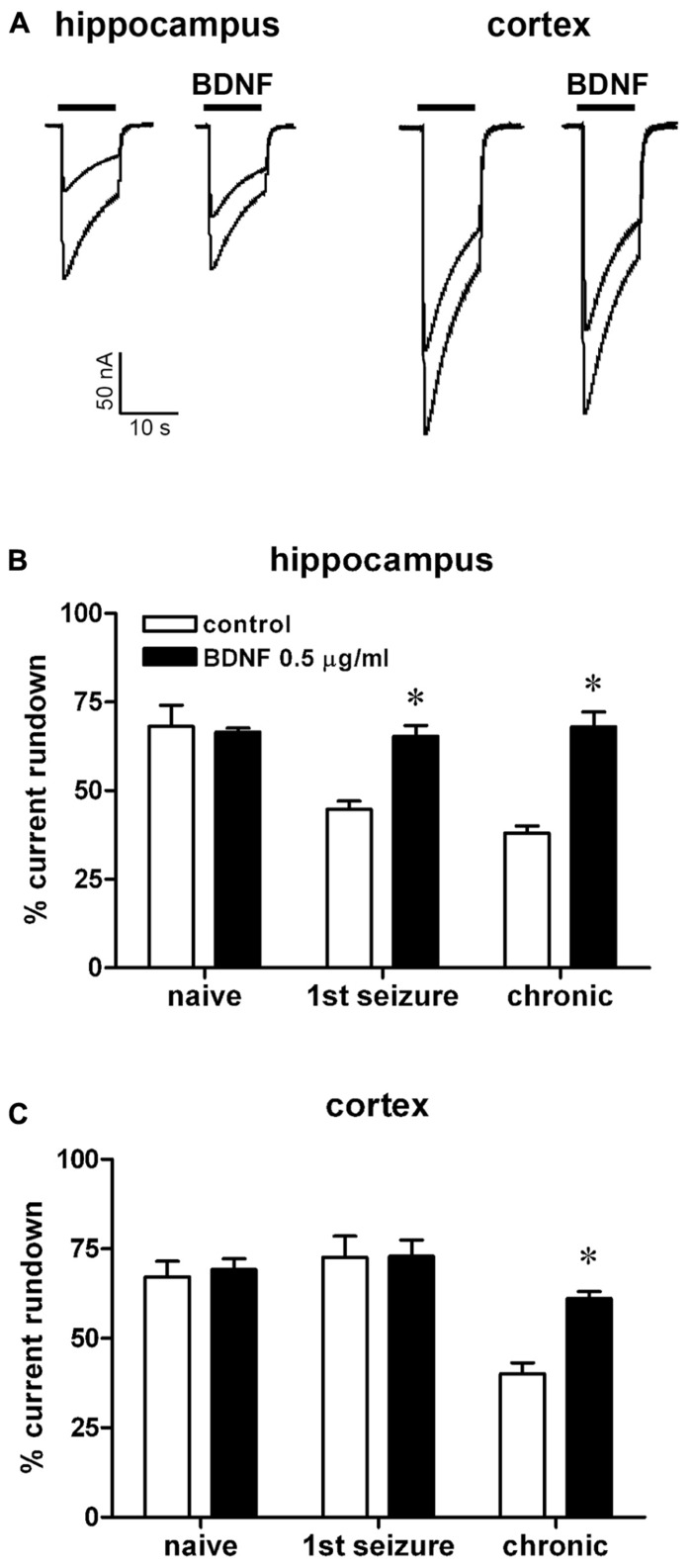
** Effect of BDNF on *I*_GABA_ run-down from oocytes injected with membranes prepared from rats killed at various time points after pilocarpine-induced SE.** **(A)** Representative superimposed currents elicited by the first and sixth GABA application (1 mM, horizontal bar) in oocytes injected with hippocampal or cortical membranes prepared from rats killed 6 h after the first spontaneous seizure, in the presence or absence of 0.5 μg/ml BDNF, as indicated. *I*_GABA_ rundown in oocytes injected with hippocampal **(B)** or cortical **(C)** membranes, in the absence or in the presence of BDNF, as indicated. Data in **(B,C)** are the means ± SEM of 15–36 oocytes per group (three to four rats; nine frogs). *I*_GABA_ peak values were normalized to the first *I*_GABA_ peak current amplitude. Holding potential, -60 mV. *P < 0.05 vs. control values, ANOVA and *post hoc* Holm–Sidak test.

**FIGURE 3:**
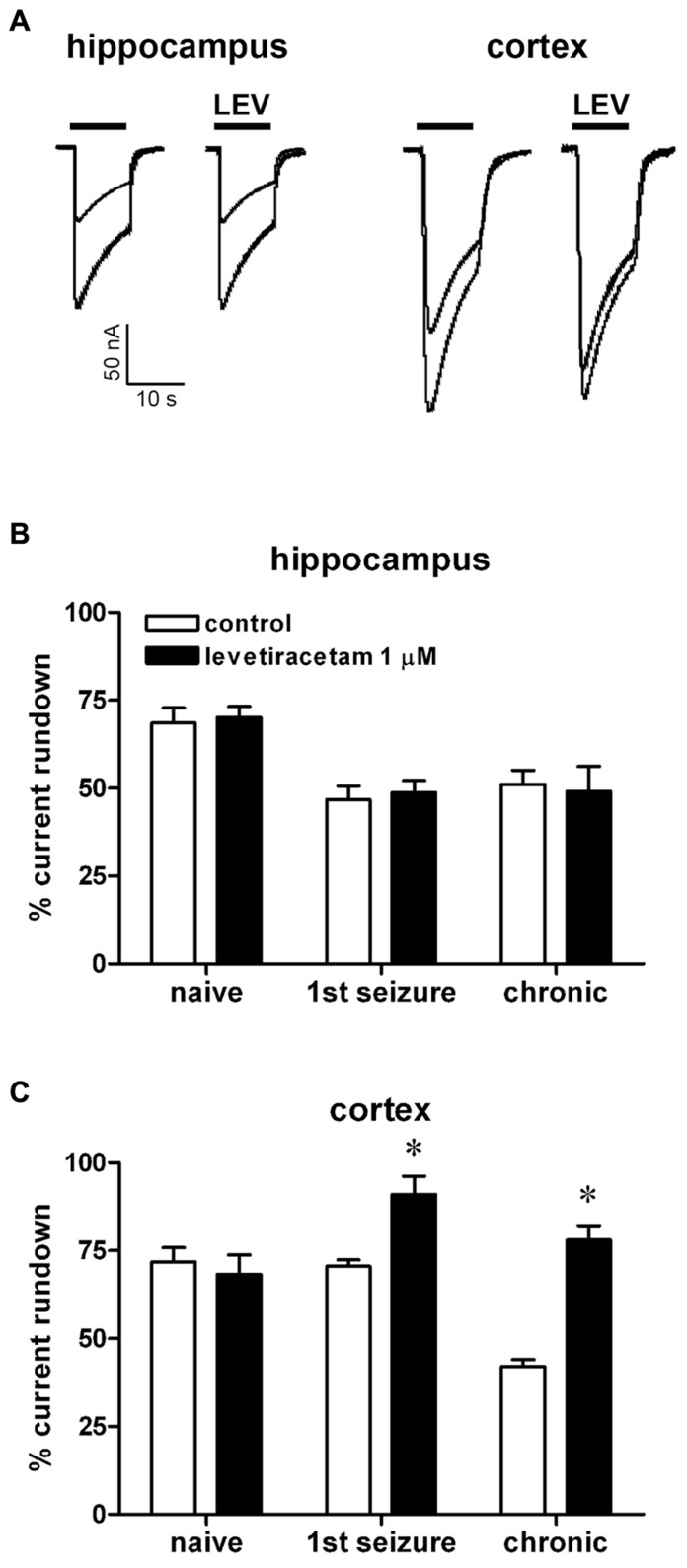
** Effect of levetiracetam (LEV) on *I*_GABA_ run-down from oocytes injected with membranes prepared from rats killed at various time points after pilocarpine-induced SE.** **(A)** Representative superimposed currents elicited by the first and sixth GABA application (1 mM, horizontal bar) in oocytes injected with hippocampal or cortical membranes prepared from rats killed 6 h after the first spontaneous seizure, in the presence or absence of 1 μM LEV, as indicated. *I*_GABA_ rundown in oocytes injected with hippocampal **(B)** or cortical **(C)** membranes, in the absence or in the presence of LEV, as indicated. Data in **(B,C)** are the means ± SEM of 9–25 oocytes per group (three to four rats; nine frogs). *I*_GABA_ peak values were normalized to the first *I*_GABA_ peak current amplitude. Holding potential, -60 mV. *P < 0.05 vs. control values, ANOVA and *post hoc* Holm–Sidak test.

All drugs were purchased from Sigma except GABA, which was purchased from Tocris (UK). Data in **Figure 1** were analyzed for fitting a single exponential curve; data in **Figures 2** and **3** were statistically analyzed using analysis of variance (ANOVA) and *post hoc* the Holm–Sidak test (SigmaPlot Software, USA).

## RESULTS

In agreement with previous reports (Palma et al., 2007b; Mazzuferi et al., 2010) applications of 1 mM GABA to oocytes injected with membranes from the cortex and hippocampus elicited inward currents that were sensitive to 100 μM bicuculline (not shown). Depending on the oocytes, the frogs and the rats, *I*_GABA_ currents had variable amplitudes: some were as large as -250 nA, others as small as -10 nA. These currents exhibited rundown after repetitive GABA applications: *I*_GABA_ elicited by the sixth GABA application fell to 69.4 ± 3 and 68.6 ± 4% of the one elicited by the first GABA application in oocytes injected with cortical and hippocampal membranes, respectively (mean ± SEM of 3 rats, 9 frogs, 49 oocytes). As previously described (Mazzuferi et al., 2010), *I*_GABA_ rundown was increased in membranes prepared from epileptic rats at the time of the first spontaneous seizure in the hippocampus (fall to 45.7 ± 3%; range 14–62%; *P* < 0.01) but not in the cortex (fall to 73.8 ± 4%; range 43–113%; **Figure 1**). Again consistent with previous reports (Mazzuferi et al., 2010), *I*_GABA_ rundown was significantly increased both in the hippocampus (44.5 ± 3%; range 20–62%; *P* < 0.01) and in the cortex (41 ± 4%; range 15–54%; *P* < 0.01) of in membranes prepared from chronic animals (**Figure 1**). This current rundown was not accompanied by a significant change in current decay and it was partially reversible after 15–20 min of washout (not shown), as previously shown in human brain tissue (Palma et al., 2004; Ragozzino et al., 2005).

Application of the neurotrophic factor BDNF abolished the increase in *I*_GABA_ rundown associated with epilepsy. Indeed, in oocytes injected with hippocampal membranes, 2-h incubation with 0.5 μg/ml BDNF decreased *I*_GABA_ rundown both in the first seizure (65.3 ± 3%) and in the chronic epilepsy group (68 ± 4%; **Figures 2A,B**). Moreover, BDNF abolished the increased *I*_GABA_ rundown in oocytes injected with cortical membranes from chronic animals (61 ± 2%), whereas it did not influence the small *I*_GABA_ rundown in first seizure animals (72.6 ± 5% **Figures 2A,C**).

The pattern of LEV effects dramatically differed from the one of BDNF. LEV (1 μM) did not affect rundown in the hippocampus, neither in control nor in epileptic tissues (48.8 ± 3 and 49.0 ± 7%, first seizure and chronic animals, respectively; **Figures 3A,B**), but significantly attenuated it in the epileptic cortex (91.0 ± 5 and 78.0 ± 4%, first seizure and chronic animals, respectively; **Figures 3A,C**). It is noteworthy that BDNF and LEV shared the same effect in decreasing *I*_GABA_ rundown only in the cortex from chronic animals. This effect was not linked to a change in the current decay (*T*_0.5_ = 9.0 ± 2.0 s in untreated cortical membranes; 8.4 ± 1.5 s with BDNF; 8.7 ± 1.0 s with LEV; *P* > 0.05).

## DISCUSSION

### MAIN FINDINGS

We found here that: (1) an increased *I*_GABA_ rundown is observed in the hippocampus but not in the cortex at the time of the first spontaneous seizure, whereas it is observed in both brain areas in the chronic period; (2) the neurotrophic factor BDNF abolishes this increased rundown in the hippocampus at the time of the first seizure, as well as in the hippocampus and cortex in the chronic period; (3) LEV does not affect rundown in the hippocampus, but attenuates it in the cortex. Below, we will discuss the possible mechanisms by which BDNF and LEV may affect *I*_GABA_ current rundown, that is, use-dependent GABA_A_ receptor desensitization; we will propose mechanisms that may underlie the alterations in rundown intensity and sensitivity to drugs during the progression of epilepsy; we will examine the implications of these findings.

### BRAIN-DERIVED NEUROTROPHIC FACTOR

The effects of BDNF in epilepsy are still controversial (Simonato et al., 2006). Whereas some studies support a proepileptogenic role (He et al., 2004), BDNF has also been reported to exert beneficial effects based on its neuroprotective and/or neurogenic actions (Paradiso et al., 2009). The anti-rundown effects of BDNF reported here confirm a previous report (Palma et al., 2007b) and suggest an anti-seizure potential. However, it is unclear why BDNF, at variance with LEV, can prevent increased GABA_A_ receptor rundown in all epileptic tissue that exhibit it, hippocampus or cortex, but does not affect rundown in normal tissue (importantly, this has been also observed in the human tissue; Palma et al., 2005b). A working hypothesis may be that this is due to modulatory effects on GABA_A_ receptor subunits expressed in the epileptic (but not as much in the normal) hippocampus and cortex.

The molecular mechanisms underlying the increased rundown in the epileptic tissue are still unknown. However, it has been hypothesized that they depend on alterations in GABA_A_ receptor subunit composition (Mazzuferi et al., 2010). Indeed, changes in the expression levels (thus, in the expected molecular composition) of GABA_A_ receptors have been described in epilepsy models and in the human epileptic tissue. Increased expression of the α 4 subunit has been reported in many studies, whereas the α 1 subunit has been reported to be slightly increased, unaltered, or even decreased (Brooks-Kayal et al., 1998; Sperk et al., 2004; Peng and Houser, 2005; Sperk, 2007). Therefore, a shift in balance toward an increase in the relative representation of α 4- compared with α 1-containing GABA_A_ receptors has been proposed, and is supported by initial immunohistochemical evidence (Mazzuferi et al., 2010). This alteration should be proepileptic because (1) the α 1 subunit is significantly more expressed in animals less susceptible to seizures, like immature (Zhang et al., 2004) or slow kindling rats (Poulter et al., 1999); (2) viral vector-mediated correction of the reduced α 1/α 4 ratio inhibits epilepsy development (Raol et al., 2006). Moreover, α 4-containing GABA_A_ receptors exhibit reduced response to repetitive GABA application, i.e., increased rundown (Lagrange et al., 2007).

It has been suggested that BDNF may favor increased α 4 gene expression and/or decreased α 1 gene expression (Brooks-Kayal and Russek, 2012). However, these effects should be pro-epileptic and, therefore, could not account for those observed in the present study. One alternative hypothesis may be based on protein kinase C (PKC) activation. It has been proposed that the abnormal GABA current run-down is caused by receptor de-phosphorylation (Palma et al., 2004) and that BDNF modulation of GABA rundown is PKC-dependent (Palma et al., 2005a). Based on these data, it may be hypothesized that BDNF corrects GABA_A_ receptor malfunction phosphorylating GABA subunits whose expression is altered in epilepsy, like the α 1 and the α 4, but also the δ or the γ2. Expression of the δ subunit has been reported to be consistently reduced in granule cell dendrites (Schwarzer et al., 1997; Sperk et al., 2004; Nishimura et al., 2005), and the δ subunits may be replaced by γ2 , resulting in impairment of both tonic and phasic GABA transmission (Zhang et al., 2007).

### LEVETIRACETAM

Levetiracetam is a widely used antiepileptic drug that also has utility in migraine prophylaxis (Lewis et al., 2004; Glauser et al., 2006). Despite its efficacy, there is no well-accepted mechanism that explains the antiepileptic action of LEV. It is well known that LEV binds to the presynaptic protein SV2A, indicating a role in vesicle exocytosis (Lynch et al., 2004). Because SV2A is implicated in maintaining the size of the readily releasable pool of synaptic vesicles (Custer et al., 2006), LEV has been suggested to directly inhibit presynaptic neurotransmitter release (Yang et al., 2007). In addition, however, PKC inhibitors have been found to block LEV effects on GABA rundown, indicating a role for PKC in LEV action (Palma et al., 2007a). LEV has been also reported to increase ROMK1 channel activity in a PKA-dependent manner (Lee et al., 2008). PKC-mediated phosphorylation of GABA_A_ receptors (with decreased rundown) and PKA-mediated phosphorylation of the ROMK1 channels (with stabilization of the resting membrane potential) may both contribute to the anti-epileptic effects of LEV, which would therefore include both a pre-synaptic (SV2A) and a post-synaptic (PKC- and PKA-dependent) component. Of course the latter and not the former may be implicated in the effect on *I*_GABA_ current rundown observed in this study.

It still remains to be determined why LEV does not reduce GABA_A_ receptor rundown in the hippocampus, whereas it reduces it in the epileptic neocortex even when it is not yet increased by the disease progression, i.e., at the time of the first spontaneous seizure. A working hypothesis may be that this is due to phosphorylation of one or more GABA_A_ subunits differentially expressed between the epileptic cortex and hippocampus. These subunit(s) should be expressed even before increased rundown is detectable in the cortex and should be different from the one that is putatively targeted by BDNF. *Ad hoc* studies should be performed to challenge this hypothesis. In any event, it is noteworthy that, in a previous work in human mTLE, LEV did not affect subicular GABA_A_ receptors whereas it profoundly influenced the cortical ones (Palma et al., 2007a), supporting the present finding that LEV effects are brain region specific.

In summary, it may be hypothesized that BDNF exerts its effects by phosphorylation of GABA subunits specifically expressed in the epileptic brain, while LEV may act on other subunit(s) that are specific to the epileptic cortex. Moreover, LEV reduces rundown in the cortex even before it is increased in the chronic epileptic period, whereas BDNF can only abolish disease-associated increases in *I*_GABA_ rundown. These observations implicate differences in efficacy on the control of seizures of different anatomical origin or occurring at different stages in the natural history of mTLE.

## CONCLUSION

In this study, we challenged the hypothesis that the disease progression affects drug responsiveness by examining the sensitivity to pharmacological treatments of the increased *I*_GABA_ rundown in the epileptic hippocampus and cortex at different stages of experimental mTLE. The data suggest that the sensitivity of GABA_A_ receptors to pharmacological interventions undergoes changes during the natural history of mTLE, implicating that site of seizure initiation and the timing of treatment may highly affect the therapeutic outcome. Further studies will be needed to better validate this hypothesis and to characterize its mechanism. These will include testing other drugs for their ability to modulate rundown in the different regions and at the different time-points, as well as analyzing the alterations in GABA receptor subunit composition during epilepsy development and correlating it with rundown. Importantly, part of these experiments is amenable to verification in the human tissue. If successful, these studies may lead to new and more effective therapies.

## Conflict of Interest Statement

The authors declare that the research was conducted in the absence of any commercial or financial relationships that could be construed as a potential conflict of interest.

## References

[B1] Brooks-Kayal A. R. Russek S. J. (2012). “Regulation of GABAA receptor gene expression and epilepsy,” in *Jasper’s Basic Mechanisms of the Epilepsies* eds Noebels J. L. Avoli M. Rogawski M. A. Olsen R. W. Delgado-Escueta A. V. (Bethesda: National Center for Bio-technology Information US) 1–9 22787609

[B2] Brooks-Kayal A. R. Shumate M. D. Jin H. Rikhter T. Y. Coulter D. A. (1998). Selective changes in single cell GABA(A) receptor subunit expression and function in temporal lobe epilepsy. *Nat. Med.* 4 1166–1172 10.1038/2661 9771750

[B3] Custer K. L. Austin N. S. Sullivan J. M. Bajjalieh S. M. (2006). Synaptic vesicle protein 2 enhances release probability at quiescent synapses. *J. Neurosci.* 26 1303–1313 10.1523/JNEUROSCI.2699-05.2006 16436618 PMC6674579

[B4] Glauser T. A. Ayala R. Elterman R. D. Mitchel W. G. Van Orman C. B. Gauer L. J. (2006). Double-blind placebo-controlled trial of adjunctive levetiracetam in pediatric partial seizures. *Neurology* 66 1654–1660 10.1212/01.wnl.0000217916.00225.3a 16641323

[B5] He X. P. Kotloski R. Nef S. Luikart B. W. Parada L. F. McNamara J. O. (2004). Conditional deletion of TrkB but not BDNF prevents epileptogenesis in the kindling model. *Neuron* 43 31–42 10.1016/j.neuron.2004.06.019 15233915

[B6] Lagrange A. H. Botzolakis E. J. Macdonald R. L. (2007). Enhanced macroscopic desensitization shapes the response of alpha4 subtype-containing GABAA receptors to synaptic and extrasynaptic GABA. *J. Physiol.* 578 655–676 10.1113/jphysiol.2006.122135 17124266 PMC2151343

[B7] Lee C. H. Lee C. Y. Tsai T. S. Liou H. H. (2008). PKA-mediated phosphorylation is a novel mechanism for levetiracetam, an antiepileptic drug, activating ROMK1 channels. *Biochem. Pharmacol.* 76 225–235 10.1016/j.bcp.2008.04.012 18547545

[B8] Lewis D. Ashwal S. Hershey A. Hirtz D. Yonker M. Silberstein S. (2004). Practice parameters: pharmacological treatment of migraine headache in children and adolescent: report of the American Academy of Neurology Quality Standards Subcommittee and the Practice Committee of the child Neurology Society. *Neurology* 63 2215–2224 10.1212/01.WNL.0000147332.41993.90 15623677

[B9] Lynch B. A. Lambeng N. Nocka K. Kensel-Hammes P. Bajjalieh S. M. Matagne A. (2004). The synaptic vesicle protein SV2A is the binding site for the antiepileptic drug levetiracetam. *Proc. Natl. Acad. Sci. U.S.A.* 101 9861–9866 10.1073/pnas.0308208101 15210974 PMC470764

[B10] Mazzuferi M. Palma E. Martinello K. Maiolino F. Roseti C. Fucile S. (2010). Enhancement of GABA(A)-current run-down in the hippocampus occurs at the first spontaneous seizure in a model of temporal lobe epilepsy. *Proc. Natl. Acad. Sci. U.S.A.* 107 3180–3185 10.1073/pnas.0914710107 20133704 PMC2840298

[B11] Miledi R. Palma E. Eusebi F. (2006). Microtransplantation of neurotransmitter receptors from cells to *Xenopus* oocyte membranes: new procedure for ion channel studies. *Methods Mol. Biol.* 322 347–355 10.1007/978-1-59745-000-3_24 16739735

[B12] Nishimura T. Schwarzer C. Gasser E. Kato N. Vezzani A. Sperk G. (2005). Altered expression of GABA(A) and GABA(B) receptor subunit mRNAs in the hippocampus after kindling and electrically induced status epilepticus. *Neuroscience* 134 691–704 10.1016/j.neuroscience.2005.04.013 15951123

[B13] Palma E. Ragozzino A. Di Angelantonio S. Spinelli G. Trettel F. Martinez-Torres A. (2004). Phosphatase inhibitors remove the run-down of gamma-aminobutyric acid type A receptors in the human epileptic brain. *Proc. Natl. Acad. Sci. U.S.A.* 101 10183–10188 10.1073/pnas.0403683101 15218107 PMC454185

[B14] Palma E. Ragozzino D. Di Angelantonio S. Mascia A. Maiolino F. Manfredi M. (2007a). The antiepileptic drug levetiracetam stabilizes the human epileptic GABAA receptors upon repetitive activation. *Epilepsia* 48 1842–1849 10.1111/j.1528-1167.2007.01131.x 17521347

[B15] Palma E. Roseti C. Maiolino F. Fucile S. Martinello K. Mazzuferi M. (2007b). GABAA-current rundown of temporal lobe epilepsy is associated with repetitive activation of GABAA “phasic” receptors. *Proc. Natl. Acad. Sci. U.S.A.* 104 20944–20948 10.1073/pnas.0710522105 18083839 PMC2409246

[B16] Palma E. Spinelli G. Torchia G. Martinez-Torres A. Ragozzino D. Miledi R. (2005a). Abnormal GABAA receptors from the human epileptic hippocampal subiculum microtransplanted to *Xenopus* oocytes. *Proc. Natl. Acad. Sci. U.S.A.* 102 2514–2518 10.1073/pnas.0409687102 15695331 PMC549013

[B17] Palma E. Torchia G. Limatola C. Trettel F. Arcella A. Cantore G. (2005b). BDNF modulates GABAA receptors microtransplanted from the human epileptic brain to *Xenopus* oocytes. *Proc. Natl. Acad. Sci. U.S.A.* 102 1667–1672 10.1073/pnas.0409442102 15665077 PMC547850

[B18] Paradiso B. Marconi P. Zucchini S. Berto E. Binaschi A. Bozac A. (2009). Localized delivery of fibroblast growth factor-2 and brain-derived neurotrophic factor reduces spontaneous seizures in an epilepsy model. *Proc. Natl. Acad. Sci. U.S.A.* 106 7191–7196 10.1073/pnas.0810710106 19366663 PMC2678472

[B19] Paradiso B. Zucchini S. Su T. Bovolenta R. Berto E. Marconi P. (2011). Localized overexpression of FGF-2 and BDNF in hippocampus reduces mossy fiber sprouting and spontaneous seizures up to 4 weeks after pilocarpine-induced status epilepticus. *Epilepsia* 52 572–578 10.1111/j.1528-1167.2010.02930.x 21269288

[B20] Peng Z. Houser C. R. (2005). Temporal patterns of fos expression in the dentate gyrus after spontaneous seizures in a mouse model of temporal lobe epilepsy. *J. Neurosci.* 25 7210–7220 10.1523/JNEUROSCI.0838-05.2005 16079403 PMC6725230

[B21] Pitkänen A. Lukasiuk K. (2011). Mechanisms of epileptogenesis and potential treatment targets. *Lancet Neurol.* 10 173–186 10.1016/S1474-4422(10)70310-0 21256455

[B22] Pitkänen A. Sutula T. P. (2002). Is epilepsy a progressive disorder? Prospects for new therapeutic approaches in temporal-lobe epilepsy. *Lancet Neurol.* 1 173–181 12849486 10.1016/s1474-4422(02)00073-x

[B23] Poulter M. O. Brown L. A. Tynan S. Willick G. William R. McIntyre D. C. (1999). Differential expression of alpha1, alpha2, alpha3 and alpha5 GABAA receptor subunits in seizure-prone and seizure-resistant rat model of temporal lobe epilepsy. *J. Neurosci.* 19 4654–4661 10341263 10.1523/JNEUROSCI.19-11-04654.1999 PMC6782587

[B24] Racine R. J. (1972). Modification of seizure activity by electrical stimulation. *II. Motor seizure. Electroencephalogr. Clin. Neurophysiol.* 32 281–294 10.1016/0013-4694(72)90177-0 4110397

[B25] Ragozzino D. Palma E. Di Angelantonio S. Amici M. Mascia A. Arcella A. (2005). Run-down of GABAA-receptors is a dysfunction associated with human temporal lobe epilepsy lacking temporal cortex lesions. *Proc. Natl. Acad. Sci. U.S.A.* 102 15219–15223 10.1073/pnas.0507339102 16217016 PMC1257725

[B26] Raol Y. H. Lund I. V. Bandyopadhyay S. Zhang G. Roberts D. S. Wolfe J. H. (2006). Enhancing GABA(A) receptor alpha 1 subunit levels in hippocampal dentate gyrus inhibits epilepsy development in an animal model of temporal lobe epilepsy. *J. Neurosci.* 26 11342–11346 10.1523/JNEUROSCI.3329-06.2006 17079662 PMC6674546

[B27] Schmidt D. Löscher W. (2005). Drug resistance in epilepsy: putative neurobiologic and clinical mechanisms. *Epilepsia* 46 858–877 15946327 10.1111/j.1528-1167.2005.54904.x

[B28] Schwarzer C. Tsunashima K. Wanzenböck C. Fuchs K. Sieghart W. Sperk G. (1997). GABA(A) receptor subunits in the rat hippocampus II: altered distribution in kainic acid-induced temporal lobe epilepsy. *Neuroscience* 80 1001–1017 9284056 10.1016/s0306-4522(97)00145-0

[B29] Simonato M. Tongiorgi E. Kokaia M. (2006). Angels and demons: neurotrophic factors and epilepsy. *Trends Pharmacol. Sci.* 27 631–638 10.1016/j.tips.2006.10.002 17055067

[B30] Sperk G. (2007). Changes in GABAA receptors in status epilepticus. *Epilepsia* 48 (Suppl. 8) 11–13 10.1111/j.1528-1167.2007.01336.x 18329986 PMC3034838

[B31] Sperk G. Furtinger S. Schwarzer C. Pirker S. (2004). GABA and its receptors in epilepsy. *Adv. Exp. Med. Biol.* 103 548–592 10.1007/978-1-4757-6376-8_7 15250588

[B32] Williams P. A. White A. M. Clark S. Ferraro D. J. Swiercz W. Staley K. J. (2009). Development of spontaneous recurrent seizures after kainate-induced status epilepticus. *J. Neurosci.* 29 2103–2112 10.1523/JNEUROSCI.0980-08.2009 19228963 PMC2897752

[B33] Yang X. F. Weisenfeld A. Rothman S. M. (2007). Prolonged exposure to levetiracetam reveals a presynaptic effect on neurotransmission. *Epilepsia* 48 1861–1869 10.1111/j.1528-1167.2006.01132.x 17521346

[B34] Zhang G. Raol Y. H. Hsu F. C. Coulter D. A. Brooks-Kayal A. R. (2004). Effects of status epilepticus on hippocampal GABAA receptors are age-dependent. *Neuroscience* 125 299–303 10.1016/j.neuroscience.2004.01.040 15062973 PMC2441871

[B35] Zhang N. Wei W. Mody I. Houser C. R. (2007). Altered localization of GABA(A) receptor subunits on dentate granule cell dendrites influences tonic and phasic inhibition in a mouse model of epilepsy. *J. Neurosci.* 27 7520–7531 10.1523/JNEUROSCI.1555-07.2007 17626213 PMC6672608

